# Neural Network Prediction of Corn Stover Saccharification Based on Its Structural Features

**DOI:** 10.1155/2018/9167508

**Published:** 2018-08-12

**Authors:** Le Gao, Shulin Chen, Dongyuan Zhang

**Affiliations:** Tianjin Key Laboratory for Industrial Biological Systems and Bioprocessing Engineering, Tianjin Institute of Industrial Biotechnology, Chinese Academy of Sciences, Tianjin, China

## Abstract

The classic assay for a large population biomass is time-consuming, labor intensive, and chemically expensive. This paper would find out a rapid assay for predicting biomass digestibility from biomass structural features without hydrolysis. We examined the 62 representative corn stover accessions that displayed a diverse cell-wall composition and varied biomass digestibility. Correlation analysis was firstly to detect effects of cell-wall compositions and wall polymer features on corn stover digestibility. Based on the dependable relationship of structural features and digestibility, a neural networks model has been developed and successfully predicted the corn stover saccharification based on the features without enzymatic hydrolysis. The actual measured and net-simulated predicted corn stover saccharification had good results as mean square error of 1.80E-05, coefficient of determination of 0.942 and average relative deviation of 3.95. The trained networks satisfactorily predicted the saccharification results based on the features of corn stover. Predicting the corn stover saccharification without hydrolysis will reduce capital and operational costs for corn stover purchasing and storage.

## 1. Introduction

Bioethanol production from lignocellulosic materials has drawn worldwide attention due to the concern about depletion of fossil fuel. In China, corn stover is one of the most common agricultural residues and can be used as feedstock to produce fuel ethanol because of its abundance, high carbohydrate content, and low cost.

As the second generation of biofuels, corn stover conversion into bioethanol principally involves three major steps: physical and chemical pretreatments for cell-wall disassociation, enzymatic digestion towards soluble sugar release, and yeast fermentation resulting in ethanol production [1]. In this process, corn stover saccharification is the critical step due to its complex structures and recalcitrance [2]. Many factors such as cell-wall compositions, wall network styles, and wall polymer features affect the corn stover digestibility [3]. For example, plant cell walls are mainly composed of cellulose, hemicelluloses, and lignin. Cellulose is a long chain of glucose molecules linked to one another primarily by glycosidic bonds [4]. Cellulose makes up about 30% of the dry mass of primary cell walls and up to 40% of the secondary cell walls. The hydrogen bonds between different layers of polysaccharides and the van der Waals forces between the parallel chains contribute to the crystalline structure of cellulose. The cellulose crystalline regions alternate with amorphous regions [5, 6]. The crystalline index (CrI) and hydrogen-bond intensity (HBI) have been characterized as the major features that affect biomass enzymatic hydrolysis in plants [7]. Lignin is associated with cellulose or hemicellulose to form a cell-wall network that is extremely recalcitrant for enzyme penetration and degradation [8]. Lignin is composed of three major phenolic components:* p*-coumaryl alcohol (H), coniferyl alcohol (G), and sinapyl alcohol (S) [9]. The efficiency of biomass saccharification during biofuel production is strongly affected not only by the total amount of lignin but also by the lignin monomer composition in plants [10].

Because of the heterogeneous nature of corn stover and many factors affecting corn stover hydrolysis, corn stover digestibility only can be measured by saccharification with a high enzyme loading for at least 72 h. It would require a significant resource investment in order to analyze a large number of samples. Therefore, the classic assay for corn stover digestibility is time-consuming, labor intensive, and chemically expensive and appears to be unsuitable for screening of large population samples [11]. Therefore, development of a rapid prediction of corn stover digestibility based on cell-wall features became more imperative. Understanding lignocellulosic features and their effects on corn stover saccharification is scientifically important for the prediction model development. This study exploited the relationship between the key determinants of plant walls and corn stover enzymatic digestion. In this paper, an artificial neural network (ANN) has been developed and successfully predicted the corn stover saccharification based on the corn stover features without enzymatic hydrolysis. This was accomplished by supplying the networks with both inputs (i.e., biomass structural features) and outputs (i.e., experimental measured saccharification from 62 corn stover samples). Successfully predicting biomass digestibility from structural features is highly valuable for the rapid assessment corn stovers, thus reducing the saccharification cost. This work will provide a way for the pricing of corn stover purchasing and storage in the future of biomass energy industry.

## 2. Material and Method

### 2.1. Plant Materials

The corn stover samples were typically selected from accessions collected in China. The samples were harvested from an experimental field in Jinan. The mature stem tissues were collected and dried tissues were ground through a 60-mesh screen and stored in a dry container until use.

### 2.2. Plant Cell-Wall Components Analysis

The cellulose and hemicelluloses contents of corn stover were quantitatively analyzed according to the NREL Laboratory Analytical Procedures (NREL, 2006) for biomass using a two-step acid method [12]. Glucan and xylan contents were calculated according to (1) and (2), where factors of 0.9 and 0.88 reflect the weight loss in converting glucose into glucan and xylose into xylan, respectively [13]. Acid-soluble lignin and acid-insoluble lignin were determined according to Chinese standard methods [13].

(1) Glucan  content  %=Glucose  released  from  acid  hydrolysis  mg×0.9Samples  weight  mg×100%.

(2) Xylan  content%=Xylose  released  from  acid  hydrolysis  mg×0.88Samples  weightmg×100%

### 2.3. FT-IR Spectroscopy and X-Ray Diffraction (XRD) Analysis

The sample was dried at -20°C at 24 h by vacuum dryer (FD-IC-50, Beijing). Infrared spectra were determined using an FT-IR 710 infrared spectrophotometer (Nicolet, Madison, WI). A total of 100 scans with a 2 cm^−1^ resolution were signal-averaged and stored; the wave number range scanned was 4000-400 cm^−1^.The ratio of absorbance at 4000-2995 cm^−1^ to those at 1337 cm^−1^ of C-OH in-plane stretching was introduced as empirical criterion of hydrogen-bond intensity (HBI) [13].

(3) $$\begin{array}{c} HBI=\frac{Absorbance\;\;\left(4000\text{ - }2995\;\;cm\text{-}1\right)}{Absorbance\;\;\left(1337cm\text{-}1\right)}. \end{array}$$

The crystallinity of samples was examined by XRD measurements performed on a Bruker D8 Advance Diffractometer using Cu K*α* radiation (*λ*=0.1541 nm) at 30 kV and 30 mA. The sample was scanned, and the intensity was recorded in 2*θ* range from 10 to 80°.

To compare the intensity difference and determine the pretreatment effect, the CrI of the corn stover was calculated by referring to the diffraction intensities of the crystalline area and amorphous region using the following:

(4) $$\begin{array}{c} CrI=\frac{I_{002}-I_{am}}{I_{002}}. \end{array}$$

### 2.4. Fourier Transform Raman (FT-Raman) Spectroscopy

To evaluate S/G ratios by FT-Raman spectroscopy, a recently developed spectral deconvolution method was used [14]. Raman spectra were collected from samples using a Bruker MultiRAM FT-Raman spectrometer with 1064 nm excitation (Bruker Optics, Inc., Billerica, MA). Laser power of 50 mW and scan number of 256 were used at a spectral resolution of 4 cm^−1^. The acquired spectra were mildly smoothed and the spectral range of 1220–1530 cm^−1^ was selected and baseline corrected using OPUS software (Bruker Optics, Inc.). The spectra were then deconvoluted at medium sensitivity using OMNIC software (Thermo Fisher Scientific, Inc., Waltham, MA). For each spectrum, S/G and H/G ratios were calculated as intensity ratio of the resolved target peaks (1331 cm^−1^ for S, 1270 cm^−1^ for G, and 1215 cm^−1^ for H) [14, 15].

### 2.5. Analysis of Biomass Enzymatic Digestibility

The biomass was subjected to the enzymatic hydrolysis by cellulase at 50°C for 72 h in triplicate. Hydrolysis experiments were conducted in 50 mL Erlenmeyer flasks with a total working volume of 20 mL while maintaining the substrate concentration of 5% (w/v). The enzyme loading was 20 FPU/g substrate. 0.5% NaN_3_ was added To the reaction mixtures to prevent microbial contamination. The samples were removed at regular intervals, and the supernatants were boiled to denature the enzyme activity and filtered through a 0.22 *μ*m filter for glucose content analysis. After hydrolysis was completed, the residues were separated from liquid by centrifugation, decantation, and filtration. Glucose in enzymatic-hydrolysis liquor was measured by high performance liquid chromatography (HPLC) (Shimadzu, Kyoto, Japan) with a refractive index detector (Shimadzu) on an Aminex HPX-87H column (Bio-Rad, Hercules, CA, USA) run at a flow rate of 0.6 mL/min at 60°C, with 5 mM H_2_SO_4_ as mobile phase [13].

### 2.6. Statistical Calculation of Correlation Coefficients

Correlation coefficients were generated by performing regression analysis for all pairs of measured traits across the whole population. The analysis used average values calculated from all original determinations for a given traits pair.

### 2.7. Artificial Neural Network

An artificial neural network, analogous to the behavior of biological neural structure, is an effective empirical modeling tool in approximating nonlinear functions, pattern recognition, and classification problems [16]. Neural networks perform the correlation without requiring a mathematical description of how the output depends on the input, which gives neural networks a key advantage over traditional approaches to function estimation. Instead, neural networks learn from examples of input-output data sets supplied to them [17].

**Figure 1:**
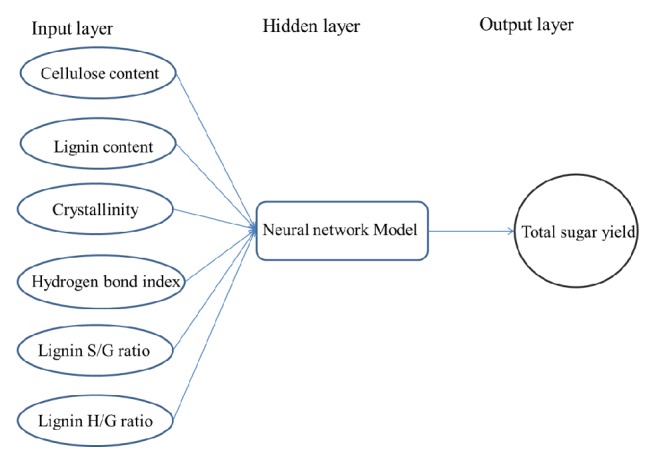
General framework of the model-based approach used in neural network model and to optimize the enzymatic-hydrolysis results of corn stover.

The fitting of the experimental data was performed in MATLAB using the neural network toolbox available in MATLAB (MathWorks, Natick, MA, USA). A multilayer feed-forward backpropagation neural network was the framework chosen for 18 networks. A neural network is an array of nodes linked by connections. The neural network model in this paper was the general regression neural network (GRNN). GRNN was a form of ANN. This GRNN creates a multilayer network. The first layer has radbas neurons and calculates weighted inputs with dist and net input with netprod. The second layer has purelin neurons and calculates weighted input with normprod and net inputs with netsum. There are six neurons in the input layer, namely, cellulose content, lignin content, CrI, HBI, S/G, and H/G. Hidden layers are employed to perform complex and nonlinear functions on the network (Figure 1). The neurons number in the hidden layer was the sample number. The relative weight of each input factor was the transposition of each input value. Lower values of MSE indicate better suitability of the model. After correct simulation on test points based on the MSE and correlation coefficient (R^2^), training was then performed on all data. After training the ANN using the training data set, validation data was used to evaluate the performance of the training based on the ability to correctly predict/simulate the validation data. The total sugar yield released from corn stover was used in the output layer. The data set used for training the GRNN model contains 62 input/output patterns. We simplified the modeling process. 76% of all samples are taken up for training and 24% of all samples for testing the model. The goal of training a network is to minimize the error between the actual outputs and the network outputs, called a training algorithm. The network outputs are compared to the actual target values until the square error is satisfied.

The mean square error (MSE) was minimized by making adjustments to the network parameters, namely, error goal, maximum number of iterations, validation checks, etc. In order to further evaluate the prediction performance, mean square error (MSE) coefficient of determination (R^2^) and average relative deviation (ARD) were utilized as the index of the prediction error of a batch:

(5) MSE=1n·∑i=1nactual  determined  valuei−predicted  valuei2.ARD=100n×∑i=1npredicted  valuei−actual  determined  valueiactual  determined  valueiR2=1−∑i=1npredicted  valuei−actual  determined  valuei2∑i=1npredicted  valuei−actual  determined  value¯2

Lower values of MSE indicate better suitability of the model. Figure 1 shows the general framework of the neural network model used in this study. The cellulose content, lignin content, CrI, HBI, S/G, and H/G were taken as the input vectors to the model, whereas the total sugar yield released from corn stover were the output vectors. The purpose of developing such a model is to obtain the optimum sugar yield upon varying input parameters.

## 3. Results and Discussion

### 3.1. Analysis of Cell-Wall Composition in Corn Stover

**Figure 2:**
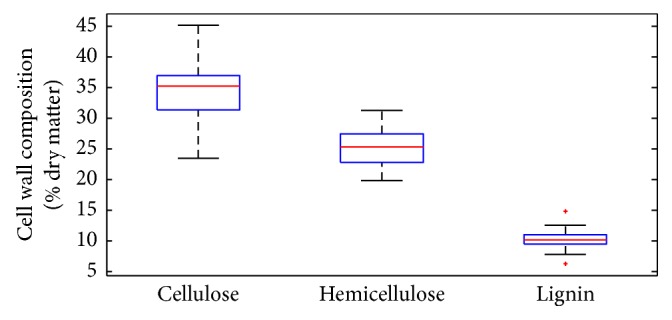
Variation of cell-wall composition of corn stover samples (n=62). The line and square in the box presented the median and mean values of all data; the bottom and top edges of the box indicated 25 and 75 percentiles of all data, respectively; and the bottom and top bars presented maximum and minimum values of all data, respectively.

Considering natural corn stover accessions include various ecological types and genetic germplasms, 62 representative corn stover samples that showed a large variation of plant cell-wall composition were selected. The cell-wall polymer composition of corn stover was analyzed (Figure 2). A diverse cell-wall composition (cellulose, hemicellulose, and lignin) was observed between different corn stover samples. The coefficient of variation (CV) values for cellulose, hemicellulose, and lignin were 21.67%, 11.47%, and 8.53%, respectively. The cellulose content of 62 corn stover samples were ranging from 23.50% to 45.17% (% dry matter), hemicellulose ranging from 19.86% to 31.33%, lignin ranging from 6.29% to 14.82%. The contents of cellulose, hemicellulose, and lignin also were significantly different. The large variation on cell-wall polymer composition offers a possibility of analyzing correlation of cell-wall composition with biomass saccharification. A previously described [18] biomass saccharification was defined by accounting the total sugar yield (hexose and pentose / dry weight) from enzymatic hydrolysis. In this current study, we determined the total sugar yield released from enzymatic hydrolysis in the total 62 corn stover accessions. The selected corn stover samples showed a great variation of biomass saccharification. The diversity of corn stover and saccharification can provide the possibility for analysis of the effects of cellulose features on biomass saccharification.

### 3.2. Effects of Cell-Wall Composition on Corn Stover Enzymatic Digestibility

**Figure 3:**
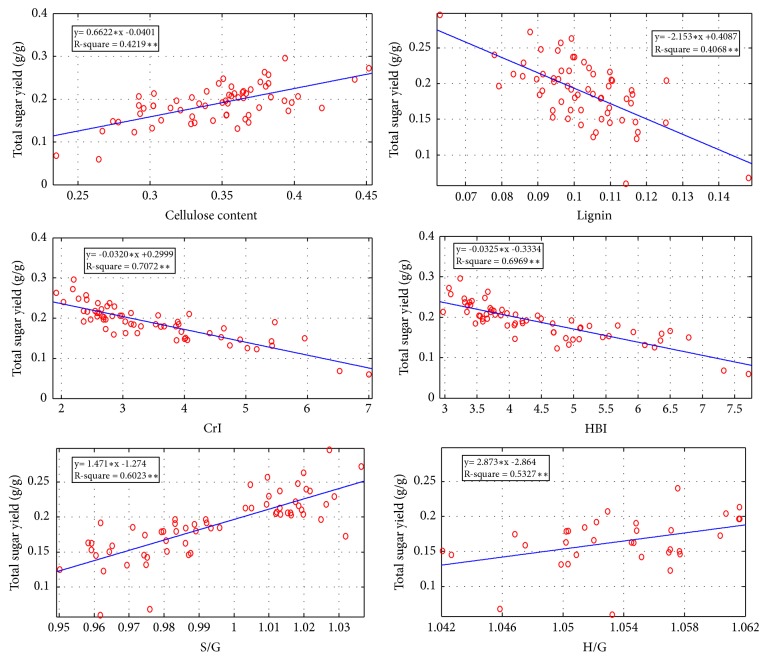
Correlation analysis between cell-wall composition (cellulose content, lignin content) / cell-wall features (CrI, HBI, S/G, H/G) of corn stover and enzymatic saccharification (n=62).

Due to the diverse compositions of plant cell walls, the 62 corn stover samples exhibited largely varied biomass digestibility. Correlation analysis was performed between cellulose and lignin content from 62 corn stover samples and enzymatic saccharification rate (Figure 3). As a result, the cellulose level showed positive correlation with the glucose yield from 62 corn stovers. The correlation R^2^ value between the cellulose content and corn stover saccharification was 0.4219, while the correlation R^2^ value between the lignin content and corn stover saccharification was 0.4068.

### 3.3. Effects of Wall Polymers on Corn Stover Enzymatic Digestibility

The cellulose microfibrils have both crystalline and amorphous regions, and the crystallinity is given by the relative amounts of these two regions. The major part of cellulose (around 2/3 of the total cellulose) is in the crystalline form. It was shown that cellulase readily hydrolyzes the more accessible amorphous portion of cellulose, while the enzyme is not so effective in degrading the less accessible crystalline portion. The 62 corn stover samples exhibited largely varied crystalline. A correlation analysis was performed to ascertain the distinct impacts of biomass features on biomass saccharification. CrI is customarily detected using raw biomass materials and has briefly been reported as a negative factor on biomass digestibility [19]. The correlation R^2^ values between the CrI and corn stover saccharification was 0.7072, which suggested that CrI showed a significantly negative correlation (Figure 3). Decreasing the crystallinity of corn stover could result in the increase of digestibility of lignocelluloses. Lower CrI would offer favorable access of cellulase to the substrate and higher biomass digestibility (Pei et al., 2016). This finding is consistent with several reports [20]. However, there has been also opposite results on correlation between crystallinity and enzymatic hydrolysis. Grethlein [21] pretreated hardwood and softwood by mild acid hydrolysis and determined their pore size distribution. It was shown that the crystallinity index has no relationship to the rate of hydrolysis. Kim and Holtzapple [22] found that the degree of crystallinity of corn stover slightly increased from 43% to 60% through delignification with calcium hydroxide, which was related to removal of amorphous components (lignin and hemicellulose). However, an increase in crystallinity of pretreated materials did not negatively affect the yield of enzymatic hydrolysis. Fan et al. [23] studied the effect of ball milling on surface area and crystallinity of cellulose. They observed an increase in crystallinity of cellulose by reducing the size of cellulose by milling. It is believed that recrystallization during water swelling may increase the crystallinity of highly ball-milled cellulose. There are two conflicting opinions that are caused by analytical methods for crystallinity. The crystallinity of pretreated biomass increased relatively by decrease in amorphous portion, e.g., lignin and hemicellulose. Maybe the most significant limitation is that they did not address the potential cross effects between structural features that may have occurred during pretreatment. Most pretreatments alter several structural features simultaneously. Studies that alter targeted structural features while ignoring the effect on nontargeted features may result in misleading information. Therefore the correlation between crystallinity and enzymatic hydrolysis could not be explained just by relative crystalline index. In this paper, it may be more correct to analyze the correlation between crystallinity and corn stover saccharification which was analyzed for raw corn stover.

The hydrogen-bond intensity (HBI) is a property specific to cellulose, considering the chain mobility and bond distance; the HBI of cellulose is closely related to the crystal system and degree of intermolecular regularity,* i.e*., crystallinity [24]. The correlation R^2^ values between the HBI and corn stover CrI was 0.55. Therefore, the correlation R^2^ values between the HBI and corn stover saccharification was 0.6969, which suggested that HBI, like the CrI, showed a significantly negative correlation (Figure 3). Decreasing the HBI of corn stover could result in the increase of digestibility of lignocelluloses.

### 3.4. Correlation of Monolignin with Corn Stover Saccharification

Given the structural diversity and chemical heterogeneity of lignin, evaluation lignin effect on biomass digestibility could be difficult [25]. The efficiency of corn stover saccharification is strongly affected not only by the total amount of lignin but also by the lignin monomer composition in plants. Determination of the relative abundance of the lignin monomers, particularly the S/G ratio and H/G ratio, is very important to fundamentally elucidate lignin structure [11]. The S/G and H/G have been determined by FT-Raman spectroscopy. The corn stover samples with high saccharification displayed relatively higher H/G values. The correlation R^2^ values between the H/G and corn stover saccharification was 0.5327, which suggested that H/G ratio had a possible positive correlation with corn stover saccharification (Figure 3). This result was consistent with the previous report that is the first time report of H/G as a positive factor in biomass enzymatic saccharification in wheat and rice [3]. On the other hand, although S/G has been reported as a negative factor in* Miscanthus* and other plants [18], the corn stover samples in this paper exhibited different result. The correlation R^2^ value between the S/G and corn stover saccharification was 0.6023, which suggested that S/G showed a possible positive correlation in corn stover saccharification. To understand the positive effect of S/G on corn stover saccharification, we further performed correlation analyses between S/G and CrI in the 62 corn stover accessions. Surprisingly, the corn stovers with high S/G ratios were found to have relatively higher cellulose CrI values. The possible negative correlation R^2^ value between the S/G and corn stover CrI was 0.4623. It suggested that S monomer may have a different interlinking with wall polymers. The exact crossing network between cellulose, hemicellulose, and lignin is far from clear [26]. With the knowledge now we could only tentatively speculate that the lignin monomers might be more important for cellulose-hemicellulose-lignin network and secondary cell-wall recalcitrance. The S monomer may have a different interlinking with wall polymers [18], which could reduce cellulose network.

### 3.5. Building the Neutral Network Model for Predicting

**Figure 4:**
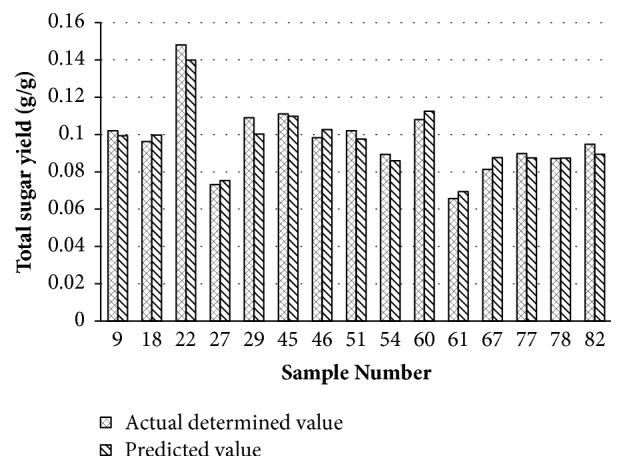
Comparison of the predicted value with the actual determined value of corn stovers saccharification.

**Figure 5:**
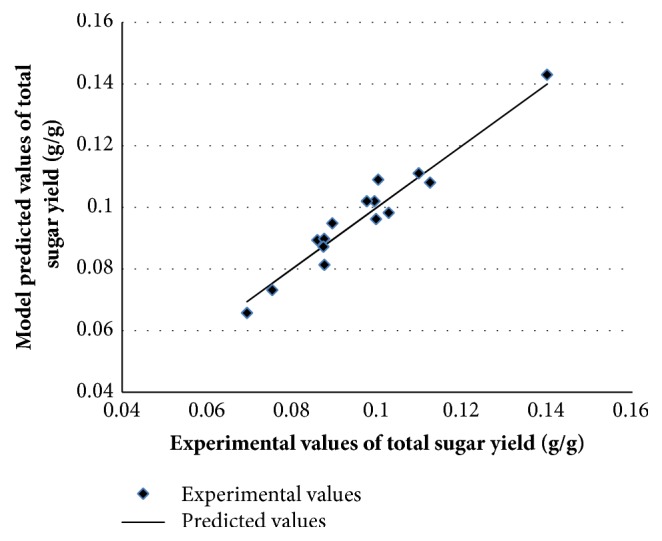
Experimental validation of model predicted total sugar yields. Comparison of neural network model predicted and experimental total sugar yields.

The 62 corn stover samples contained the following structural features: cellulose content, lignin content, crystallinity, HBI, S/G ratio, and H/G ratio, respectively. The wide spectrum of structural features made it possible to develop reliable empirical models to predict biomass digestibility from structural features. The neutral network model has been trained and built on the features of 62 corn stover samples through rolling learning-prediction approach. The selection of the most appropriate parameters for ANN modeling is considered of paramount importance for prediction of the hydrolysis process [27]. In the present work, to test the prediction capabilities of the neural network model, the predicted values obtained from the model are compared with the experimental values. The coefficient of determination (R^2^) and the average relative deviation (ARD) were 0.942 and 3.95, respectively (Table 1). The average of MSE of the net-simulated outputs of glucose released from corn stover samples features is 1.80E-05, which lies near zero. The R^2^ value of testing set was found to approach unity which confirms the reliability of the model in predicting the total sugar yield. Net-simulated outputs of total sugars released from 15 corn stover samples features were compared with measured values as shown in Figure 4. It is evident that the relative error between experimentally observed and model predicted values is very low. Performance of the model in describing the correlation between experimental and predicted total sugar yield are shown in Figure 5. The results showed that the neural network model has predictions that are closer to the line of perfect prediction. The agreement in measured and net-simulated slopes and intercepts indicated the trained networks satisfactorily predicted the saccharification results based on the features of corn stover. It has been reported in literature that ANNs are flexible as new data can be added anytime giving fitting [27].

## 4. Conclusions

Based on the structural features and saccharification of a large number of corn stover samples, a neural networks model has been developed and was demonstrated applicable for the prediction of the corn stover saccharification based on the features without enzymatic hydrolysis. The predicted value of corn stover digestibility via this model was very similar with the actual determined value of corn stover saccharification. This neural network model could offer the fast approach for bioenergy crops selection. In the future, the neural network model will have good application for corn stover storage and evaluation, which will be cost-effective and time-saving.

## Figures and Tables

**Table 1:** Performance of neural network model.

Statistical parameter	Value
R^2^	0.942
MSE	1.80E-05
ARD	3.95

## Data Availability

The data used to support the findings of this study are available from the corresponding author upon request.

## Supplementary Materials

Supplementary Materials
** Supplemental Table.1**: the compositions and wall polymer features of the samples for training.** Supplemental Table.2**: the compositions and wall polymer features of the samples for testing.

## References

[1] Rubin E. M. (2008). Genomics of cellulosic biofuels. *Nature*.

[2] Himmel M. E., Ding S., Johnson D. K. (2007). Biomass recalcitrance: engineering plants and enzymes for biofuels production. *Science*.

[3] Wu Z., Zhang M., Wang L. (2013). Biomass digestibility is predominantly affected by three factors of wall polymer features distinctive in wheat accessions and rice mutants. *Biotechnology for Biofuels*.

[4] Arioli T., Peng L. C., Betzner A. S. (1998). Molecular analysis of cellulose biosynthesis in *Arabidopsis*. *Science*.

[5] Bansal P., Hall M., Realff M. J., Lee J. H., Bommarius A. S. (2010). Multivariate statistical analysis of X-ray data from cellulose: A new method to determine degree of crystallinity and predict hydrolysis rates. *Bioresource Technology*.

[6] Park J.-Y., Kang M., Kim J. S., Lee J.-P., Choi W.-I., Lee J.-S. (2012). Enhancement of enzymatic digestibility of Eucalyptus grandis pretreated by NaOH catalyzed steam explosion. *Bioresource Technology*.

[7] Zhang Y.-H. P., Lynd L. R. (2004). Toward an aggregated understanding of enzymatic hydrolysis of cellulose: noncomplexed cellulase systems. *Biotechnology and Bioengineering*.

[8] Achyuthan K. E., Achyuthan A. M., Adams P. D. (2010). Supramolecular self-assembled chaos: Polyphenolic lignin's barrier to cost-effective lignocellulosic biofuels. *Molecules*.

[9] Ralph J., Lundquist K., Brunow G. (2004). Lignins: Natural polymers from oxidative coupling of 4-hydroxyphenyl- propanoids. *Phytochemistry Reviews*.

[10] Baucher M., Bernard-Vailhé M. A., Chabbert B. (1999). Down-regulation of cinnamyl alcohol dehydrogenase in transgenic alfalfa (*Medicago sativa* L.) and the effect on lignin composition and digestibility. *Plant Molecular Biology*.

[11] Huang J., Xia T., Li A. (2012). A rapid and consistent near infrared spectroscopic assay for biomass enzymatic digestibility upon various physical and chemical pretreatments in Miscanthus. *Bioresource Technology*.

[12] Sluiter B. H. A., Ruiz R., Scarlata C. (2008). Determination of structural carbohydrates and lignin in biomass. *NREL/TP-510-42618*.

[13] Gao L., Li D., Gao F. (2015). Hydroxyl radical-aided thermal pretreatment of algal biomass for enhanced biodegradability. *Biotechnology for Biofuels*.

[14] Sun L., Varanasi P., Yang F., Loqué D., Simmons B. A., Singh S. (2012). Rapid determination of syringyl: Guaiacyl ratios using FT-Raman spectroscopy. *Biotechnology and Bioengineering*.

[15] Papa G., Varanasi P., Sun L. (2012). Exploring the effect of different plant lignin content and composition on ionic liquid pretreatment efficiency and enzymatic saccharification of Eucalyptus globulus L. mutants. *Bioresource Technology*.

[16] Giustolisi O. (2004). Sparse solution in training artificial neural networks. *Neurocomputing*.

[17] O'Dwyer J. P., Zhu L., Granda C. B., Chang V. S., Holtzapple M. T. (2008). Neural network prediction of biomass digestibility based on structural features. *Biotechnology Progress*.

[18] Xu N., Zhang W., Ren S. (2012). Hemicelluloses negatively affect lignocellulose crystallinity for high biomass digestibility under NaOH and H2SO4 pretreatments in Miscanthus. *Biotechnology for Biofuels*.

[19] Zhang W., Yi Z., Huang J. (2013). Three lignocellulose features that distinctively affect biomass enzymatic digestibility under NaOH and H2SO4 pretreatments in Miscanthus. *Bioresource Technology*.

[20] Grohmann K., Mitchell D. J., Himmel M. E., Dale B. E., Schroeder H. A. (1989). The role of ester groups in resistance of plant cell wall polysaccharides to enzymatic hydrolysis. *Applied Biochemistry and Biotechnology*.

[21] Grethlein H. E. (1985). The effect of pore size distribution on the rate of enzymatic hydrolysis of cellulosic substrates. *Bio/Technology*.

[22] Kim S., Holtzapple M. T. (2006). Effect of structural features on enzyme digestibility of corn stover. *Bioresource Technology*.

[23] Fan M.-Q., Xu F., Sun L.-X. (2007). Studies on hydrogen generation characteristics of hydrolysis of the ball milling Al-based materials in pure water. *International Journal of Hydrogen Energy*.

[24] Gaur R., Agrawal R., Kumar R. (2015). Evaluation of recalcitrant features impacting enzymatic saccharification of diverse agricultural residues treated by steam explosion and dilute acid. *RSC Advances*.

[25] Pei Y., Li Y., Zhang Y. (2016). G-lignin and hemicellulosic monosaccharides distinctively affect biomass digestibility in rapeseed. *Bioresource Technology*.

[26] Scheller H. V., Ulvskov P. (2010). Hemicelluloses. *Annual Review of Plant Biology*.

[27] Gama R., Van Dyk J. S., Burton M. H., Pletschke B. I. (2017). Using an artificial neural network to predict the optimal conditions for enzymatic hydrolysis of apple pomace. *3 Biotech*.

