# Supplementary material for: Neural Network Prediction of Corn Stover Saccharification Based on Its Structural Features
Source: Biomed Res Int. 2018 Aug 12;2018:9167508. doi: 10.1155/2018/9167508 (PMC6109571; doi:10.1155/2018/9167508)
Supplement: Supplementary Materials — Supplemental Table.1: the compositions and wall polymer features of the samples for training. Supplemental Table.2: the compositions and wall polymer features of the samples for testing. [file 9167508.f1.docx]

|  | Cellulose | Lignin | H/G | S/G | HBI | CrI |
| --- | --- | --- | --- | --- | --- | --- |
| 1 | 0.302626581 | 0.1053 | 1.071974 | 1.013102 | 2.972092 | 3.129014 |
| 2 | 0.307766231 | 0.0983 | 1.04206 | 0.980816 | 5.45326 | 4.021243 |
| 3 | 0.375671024 | 0.1002 | 1.05711 | 0.989133 | 4.077781 | 3.671217 |
| 4 | 0.301430027 | 0.1176 | 1.050328 | 0.974889 | 4.924914 | 4.739299 |
| 5 | 0.383741194 | 0.1103 | 1.069297 | 1.011933 | 3.871113 | 2.825466 |
| 6 | 0.361146378 | 0.1105 | 1.060715 | 1.019818 | 3.532636 | 2.580344 |
| 7 | 0.274325655 | 0.1132 | 1.056964 | 0.987592 | 4.88193 | 4.003373 |
| 8 | 0.266902178 | 0.1053 | 1.070207 | 0.950161 | 6.257043 | 5.026606 |
| 9 | 0.402646749 | 0.0956 | 1.062894 | 1.016231 | 4.097971 | 2.953978 |
| 10 | 0.234992223 | 0.1482 | 1.045854 | 0.97593 | 7.33172 | 6.525264 |
| 11 | 0.289208575 | 0.1173 | 1.057061 | 0.96267 | 4.742964 | 5.176125 |
| 12 | 0.350248053 | 0.0992 | 1.06575 | 0.961815 | 4.266188 | 3.036187 |
| 13 | 0.39590593 | 0.1157 | 1.060343 | 1.031952 | 5.09771 | 2.72052 |
| 14 | 0.292199269 | 0.0896 | 1.069777 | 1.015359 | 3.781032 | 2.98214 |
| 15 | 0.393523197 | 0.0629 | 1.082072 | 1.027313 | 3.239337 | 2.197166 |
| 16 | 0.380228996 | 0.1027 | 1.068371 | 1.009949 | 3.395991 | 2.74604 |
| 17 | 0.338151789 | 0.1161 | 1.067114 | 0.995934 | 3.476665 | 3.532837 |
| 18 | 0.292242151 | 0.1052 | 1.065839 | 0.971042 | 4.208805 | 3.12899 |
| 19 | 0.368291812 | 0.1099 | 1.050873 | 0.974281 | 5.084581 | 4.047493 |
| 20 | 0.278128346 | 0.1169 | 1.057702 | 0.98707 | 4.090903 | 4.908515 |
| 21 | 0.441803119 | 0.0955 | 1.089473 | 1.004689 | 3.30396 | 2.409857 |
| 22 | 0.339224538 | 0.0986 | 1.066098 | 1.009341 | 3.885516 | 2.360748 |
| 23 | 0.264554718 | 0.1143 | 1.053244 | 0.961728 | 7.716164 | 7.001853 |
| 24 | 0.343608906 | 0.1076 | 1.057664 | 0.964297 | 6.785816 | 5.958333 |
| 25 | 0.451728985 | 0.0878 | 1.073284 | 1.036417 | 3.063877 | 2.183174 |
| 26 | 0.30235338 | 0.0905 | 1.064106 | 0.986211 | 4.674951 | 3.907162 |
| 27 | 0.328448635 | 0.1019 | 1.05517 | 0.975207 | 6.346094 | 5.417534 |
| 28 | 0.31845637 | 0.1095 | 1.064325 | 0.991919 | 4.497506 | 2.719321 |
| 29 | 0.398137884 | 0.1039 | 1.052208 | 0.992205 | 4.967037 | 2.360814 |
| 30 | 0.366589662 | 0.0914 | 1.061619 | 1.003236 | 3.340928 | 2.582437 |
| 31 | 0.353046468 | 0.099 | 1.054509 | 0.95831 | 5.927634 | 4.412259 |
| 32 | 0.293073514 | 0.1097 | 1.052029 | 0.980545 | 6.499048 | 3.952532 |
| 33 | 0.369847333 | 0.1041 | 1.076343 | 1.017644 | 3.703975 | 2.652838 |
| 34 | 0.393119273 | 0.0792 | 1.061625 | 0.983103 | 3.659037 | 2.474349 |
| 35 | 0.314110734 | 0.1073 | 1.050344 | 0.979862 | 5.686996 | 3.295304 |
| 36 | 0.379460573 | 0.0993 | 1.072854 | 1.019943 | 3.669218 | 1.914335 |
| 37 | 0.350728749 | 0.0908 | 1.083652 | 1.018247 | 3.623264 | 2.275602 |
| 38 | 0.329011637 | 0.1255 | 1.068972 | 1.013294 | 4.439341 | 2.66307 |
| 39 | 0.329291756 | 0.1092 | 1.047508 | 0.965154 | 6.365676 | 2.851717 |
| 40 | 0.29555792 | 0.1145 | 1.05022 | 0.979208 | 5.246483 | 3.615588 |
| 41 | 0.382050802 | 0.0997 | 1.069237 | 1.021735 | 3.382232 | 2.782625 |
| 42 | 0.356448693 | 0.086 | 1.076378 | 1.02868 | 3.353283 | 2.860254 |
| 43 | 0.330950332 | 0.1253 | 1.042644 | 0.960499 | 4.98896 | 3.87322 |
| 44 | 0.364164046 | 0.1099 | 1.075072 | 1.018493 | 3.759005 | 2.420785 |
| 45 | 0.360598592 | 0.106 | 1.049868 | 0.96937 | 6.106723 | 5.429224 |
| 46 | 0.36484463 | 0.0965 | 1.076425 | 1.026399 | 3.728472 | 2.549827 |
| 47 | 0.352451649 | 0.0982 | 1.061577 | 1.024909 | 3.583808 | 2.683307 |
| 48 | 0.356448001 | 0.0943 | 1.052929 | 1.012202 | 3.646962 | 3.572111 |
| 49 | 0.376439447 | 0.078 | 1.057543 | 1.020706 | 3.422964 | 2.032244 |
| 50 | 0.364868146 | 0.0944 | 1.069377 | 1.016336 | 3.550725 | 2.690251 |
| 51 | 0.352971078 | 0.1019 | 1.050201 | 0.986099 | 4.693898 | 3.232633 |
| 52 | 0.358451711 | 0.0832 | 1.073695 | 1.004974 | 3.708606 | 2.611657 |
| 53 | 0.347548544 | 0.1001 | 1.071517 | 1.013098 | 3.312408 | 2.593467 |
| 54 | 0.366006601 | 0.0941 | 1.057058 | 0.959235 | 5.547557 | 4.595588 |
| 55 | 0.382110284 | 0.0963 | 1.089934 | 1.009572 | 3.090972 | 2.398985 |
| 56 | 0.302234416 | 0.101 | 1.051429 | 0.993455 | 4.094062 | 3.175433 |
| 57 | 0.41914813 | 0.1075 | 1.054843 | 0.983451 | 3.970968 | 3.860644 |
| 58 | 0.321144121 | 0.0965 | 1.046834 | 0.974529 | 5.110774 | 4.640911 |
| 59 | 0.353917255 | 0.1159 | 1.054799 | 0.983129 | 4.20493 | 3.893376 |
| 60 | 0.354774901 | 0.0858 | 1.076388 | 1.019368 | 3.965889 | 4.076209 |
| 61 | 0.334064831 | 0.0912 | 1.063037 | 0.98873 | 3.590817 | 5.468716 |
| 62 | 0.368287498 | 0.0941 | 1.054657 | 0.959315 | 4.685997 | 3.041131 |
